# Progressive Increase of Inflammatory CXCR4 and TNF-Alpha in the Dorsal Root Ganglia and Spinal Cord Maintains Peripheral and Central Sensitization to Diabetic Neuropathic Pain in Rats

**DOI:** 10.1155/2019/4856156

**Published:** 2019-03-14

**Authors:** Dan Zhu, Tingting Fan, Xinyue Huo, Jian Cui, Chi Wai Cheung, Zhengyuan Xia

**Affiliations:** ^1^Department of Pain Care, Southwest Hospital, Army Medical University, Chongqing 400038, China; ^2^Department of Anaesthesiology, The University of Hong Kong, HKSAR, China; ^3^Department of Biostatistics and Bioinformatics, Rollins School of Public Health, Emory University, USA

## Abstract

Diabetic neuropathic pain (DNP) is a common and serious complication of diabetic patients. The pathogenesis of DNP is largely unclear. The proinflammation proteins, CXCR4, and TNF-*α* play critical roles in the development of pain, while their relative roles in the development of DNP and especially its progression is unknown. We proposed that establishment of diabetic pain models in rodents and evaluating the stability of behavioral tests are necessary approaches to better understand the mechanism of DNP. In this study, Von Frey and Hargreaves Apparatus was used to analyze the behavioral changes of mechanical allodynia and heat hyperalgesia in streptozotocin-induced diabetic rats at different phases of diabetes. Moreover, CXCR4 and TNF-*α* of spinal cord dorsal and dorsal root ganglia (DRG) were detected by western blotting and immunostaining over time. The values of paw withdrawal threshold (PWT) and paw withdrawal latencies (PWL) were reduced as early as 1 week in diabetic rats and persistently maintained at lower levels during the progression of diabetes as compared to control rats that were concomitant with significant increases of both CXCR4 and TNF-*α* protein expressions in the DRG at 2 weeks and 5 weeks (the end of the experiments) of diabetes. By contrast, CXCR4 and TNF-*α* in the spinal cord dorsal horn did not significantly increase at 2 weeks of diabetes while both were significantly upregulated at 5 weeks of diabetes. The results indicate that central sensitization of spinal cord dorsal may result from persistent peripheral sensitization and suggest a potential reference for further treatment of DNP.

## 1. Introduction

Diabetes is an increasingly common chronic global epidemic, and relevant neuropathy is its most common and disabling complication. DNP seriously affects the quality of life [1]. Approximately 20-30% of diabetic patients develop neuropathic pain, and the major clinical manifestations are spontaneous pain, hyperalgesia, and allodynia [2, 3]. These symptoms are the consequences of peripheral (e.g., dorsal root ganglia (DRG)) and/or central (e.g., spinal cord dorsal) sensitization to noxious stimuli. In clinic, neither tight control blood glucose nor inhibition of key enzymes of glucose metabolism could completely alleviate DNP [4–6], all of which are attributed to incomplete understanding of the DNP mechanism. Clinical manifestations of peripheral and central sensitization of various pain diseases can be differentiated by consultation and physical examination [7–9]. More objective measuring instruments are needed to distinguish mechanical allodynia and heat hyperalgesia in a rodent DNP model [10, 11]. However, with the development of advanced neuropathic pain, followed by the loss of sensory function or tactile hypoesthesia [12–14], mechanical allodynia and heat hyperalgesia cannot be accurately measured by Von Frey and Hargreaves Apparatus. The limitations of behavioral test exist in the animal model were inevitable [15, 16].

Inflammation proteins, such as tumor necrosis factor-alpha (TNF-*α*), chemokine receptor type 4 (CXCR4), have been highlighted as mediators in neuropathic pain [17, 18]. These proteins perform various functions [17–20]; however, their changes in the spinal cord dorsal at relatively early and late phase of DNP are unknown. Therefore, exploring the time course of peripheral and central sensitization would help us to avoid the defects in the DNP animal model. In the present study, we analyzed the positive number proportion of mechanical allodynia and heat hyperalgesia of the DNP model in different time course. In addition, we examined the expression of inflammation protein in DRG and spinal cord dorsal at an early and late phase of streptozotocin-induced type 1 diabetic rats to illustrate the correlation of peripheral and central sensitization in the development of DNP.

## 2. Materials and Methods

### 2.1. Animals

The protocols of this research were approved by the Committee on the Use of Live Animals in Teaching and Research (CULATR) (permit number: 3995-16/3995-15). The animals were handed in accordance with the guide for the care and use of laboratory animals published by the Laboratory Animal Unit (LAU) at the University of Hong Kong. Adult Sprague-Dawley rats, weighing 220-250 g, were housed two rats per cage in an environment of 25°C of temperature with humidity of 60% under a 12 hour/12 hour light-dark cycle with ad libitum access to food and water.

### 2.2. The Establishment of the DNP Model

The diabetogenic streptozotocin- (STZ-) induced diabetes cause mechanical allodynia and heat hyperalgesia in hind paw, which is a typic DNP model [21]. Streptozotocin (Sigma, USA) intravenous injection is a commonly used and relatively simple method to induce type 1 diabetes with one-time injection in rodents [22]. Rats were anesthetized under intraperitoneal injection of ketamine 67.7 mg/kg and xylazine 6.77 mg/kg and injected with 65 mg/kg dose of STZ as we previously reported [23, 24]. After 3 days of injection, the blood glucose level was tested by glucometer (OneTouch Ultra) and blood glucose level over 16.7 mmol/L was considered as diabetes and included in the study. After 1 week of STZ injection, the mechanical allodynia and heat hyperalgesia were measured in diabetic rats and compared with nondiabetic control rats to confirm the establishment of DNP.

### 2.3. Behavioral Test

**Figure 1:**
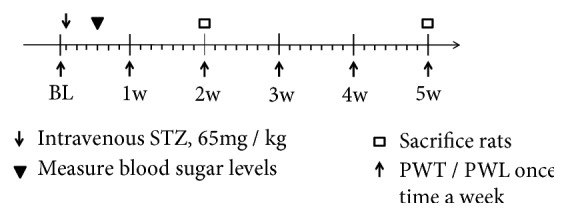
Timeline of experiment protocol. BL = baseline; w = week; PWT = paw withdrawal threshold; PWL = paw withdrawal latencies.

Behavioral tests of mechanical allodynia and heat hyperalgesia were performed, and the values obtained before intravenous STZ injection were recorded as baseline. Mechanical allodynia and heat hyperalgesia were measured over the course of 2 or 5 weeks after STZ-induced diabetes to determine the persistent development of neuropathic pain (Figure 1). DNP is a systemic disease in which the location of pain is unevenly distributed. Stocking-glove pattern is the usual presentation of diabetic neuropathy [25]. In the DNP animal model, bilateral hind paw withdrawals were selected as positive symptoms in behavioral test.

#### 2.3.1. Mechanical Allodynia

Mechanical allodynia of bilateral hind paw was tested by an electronic Von Frey (IITC/Life Science, Inc., USA) and measured as described previously [26, 27]. Briefly, the rats were numbered and placed individually in plexiglass boxes with a metal mesh floor and allowed to adapt to the environment at least 15 minutes before measurement. Blunted Von Frey filament with a range of force was involved perpendicularly onto the planter surface of hind paw. The weakest filament that can stimulate a brisk withdrawal was applied. The paw withdrawal test was repeated three times with an interval of 5 minutes for each foot, and the mean value was calculated as the mechanical threshold.

#### 2.3.2. Thermal Hyperalgesia

Thermal hyperalgesia was determined by Hargreaves Apparatus (Ugo Basile, Varese, Italy) as reported [27]. Rats were placed in a plexiglass box to accommodate to the environment 15 minutes before testing. A movable and radiant heat light source was placed under the plexiglass plate and focused on the hind paw. The latency (maximum of 20 s) of paw withdrawal (PWL) that moved away from the heat light source was recorded. To avoid heat source stimulation, a 5-minute interval between each trial was set. Each measurement was repeated 3 times, and the mean value was calculated.

### 2.4. Spinal Cord and DRG Tissue Preparation

Rats were sacrificed at 2 or 5 weeks of diabetes by an overdose of sodium pentobarbital, and the spinal cord and DRG were obtained. Bilateral L3-L5 of spinal cord and DRG were rapidly removed and stored at -80°C for protein assays with western blot. For immunofluorescence, rats were perfused with 4% paraformaldehyde, and the L3-L5 of spinal cord tissues were isolated and postfixed in 4% paraformaldehyde overnight at 4°C and then placed in 30% sucrose to be assessed by immunofluorescence staining as stated below.

### 2.5. Western Blot

Tissue samples of spinal cord were homogenized (Polytron, Kinematica, Switzerland) in 100*μ*L ice-cold lysis buffer with protease inhibitor cocktails (Sigma-Aldrich, USA). Protein concentrations were determined by the Bradford assay kit (Bio-Rad, USA). Equal amount of proteins was subjected to electrophoresis in 12.5% SDS-polyacrylamide gels and then transferred to polyvinylidene fluoride (PVDF) membranes (Bio-Rad, USA). After blocking with 5% nonfat milk for 2 h, the membranes were incubated with rabbit anti-CXCR4 (1 : 1000, Abcam, UK), rabbit anti-TNF-*α* (1 : 1000, Abcam, UK), and *β*-Tubblin (1 : 1000, Cell Signaling, USA) overnight at 4°C. After washing with 0.01 M Tris-buffered saline Tween-20 for 10 min 3 times, the membranes were incubated for 2 h at room temperature with secondary antibodies HRP-linked anti-rabbit IgG (1 : 2000, Cell Signaling, USA). Protein expressions were visualized by exposure to X-ray films (Kodak, USA) after incubation of blot in ECL (Bio-Rad, USA) and quantified by ImageJ software (National Institutes of Health, USA).

### 2.6. Immunofluorescence

20 *μ*m slices were cut by a cryotome (Thermo, USA). Slices were dried in ventilation for 30 minutes and then permeabilized by 0.25% triton-X 100 for 15 minutes. The sections were blocked with 10% bovine serum albumin (Sigma, USA) in phosphate buffer saline (PBS) diluent for 1 hour at room temperature. Then sections were incubated with rabbit monoclonal anti-CXCR4 (1 : 200, Abcam, USA) or rabbit polyclonal anti-TNF-*α* (1 : 200, Abcam) overnight at 4°C. After slices were washed with PBS for 10 minutes 3 times, added Alexa Fluor 568-conjugated anti-rabbit antibody (1 : 500, Abcam, USA) diluted in antibody dilution buffer (Dako, Denmark) for 1 h at room temperature and followed by nuclear staining using DAPI (Cell Signaling Technology, USA). Images were detected by a confocal microscope LSM 710 (Zeiss) and measured using ImageJ Software (National Institutes of Health, USA).

### 2.7. Statistical Analysis

All data are expressed as mean ± S.D. Results of western blot and immunofluorescence work were tested using one-way analysis of variance (ANOVA) followed by Tukey's post hoc test for multiple comparisons of group means. Results of Von Frey and Hargreaves Apparatus were analyzed by two-way ANOVA followed by Tukey's post hoc test. Data were analyzed with statistical software SPSS 19.0 (USA) and GraphPad Prism 7.0 (USA). *P* value < 0.05 was considered statistically significant in the study.

## 3. Results

### 3.1. Mechanical Allodynia and Heat Hyperalgesia Developed in the DNP Model

**Figure 2:**
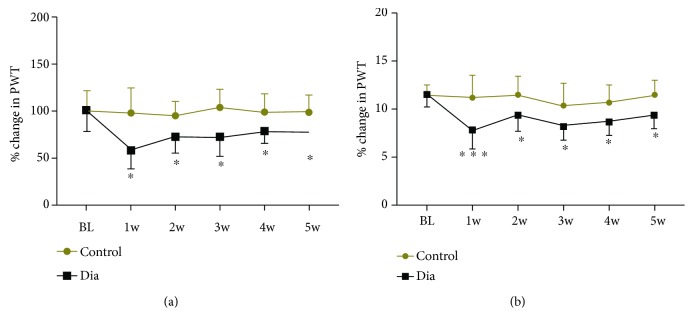
Variations of PWT (a) and PWL (b) during the progression of STZ-induced diabetes (Dia) in rats. PWT and PWL were significantly decreased from 1 week to 5 weeks as detected by electronic Von Frey and Hargreaves test. All results are presented as means ± SD, *n* = 19/group, ^∗^*P* < 0.05, ^∗∗^*P* < 0.01, ^∗∗∗^*P* < 0.001, Dia vs. control.

To explore the potential behavioral changes of DNP in STZ-induced diabetic rats, Von Frey and Hargreaves Apparatus was used to assess mechanical allodynia and heat hyperalgesia (Figures 2(a) and 2(b)). Baseline values of paw withdrawal latencies (PWL) and paw withdrawal threshold (PWT) were tested in rats in the nondiabetic control group and in rats to be used for the induction of diabetes with STZ, and no significant difference existed between the saline and diabetes group. The values of PWT and PWL decreased as early as 1 week after the induction of diabetes and maintained at a constantly lower level in the diabetes group up to 5 weeks of diabetes (the duration of the experiments) than that in the control group (all *P* < 0.05), indicative of the development of DNP. However, as shown in Table 1, the pain threshold in the diabetic group did not continuously decease over time. But instead, the pain threshold demonstrated a trend of gradual increase despite that the degree of increase did not reach statistical significance. Unexpectedly and intriguingly, the total number of diabetic rats that demonstrated DNP peaked at 1 week after diabetic induction, and the percentage of diabetic rats that had DNP decreased from 89% to 42% in PWT and from 84% to 52% in PWL. The data above indicated that the duration of diabetes, at least in STZ-induced type 1 diabetes, has significant impact on the appearance of DNP and thus the optimal establishment of the DNP model.

### 3.2. Changes of Lumbar Spinal Cord TNF-*α* and CXCR4 Protein Expression over Time in Diabetic Rats

**Figure 3:**
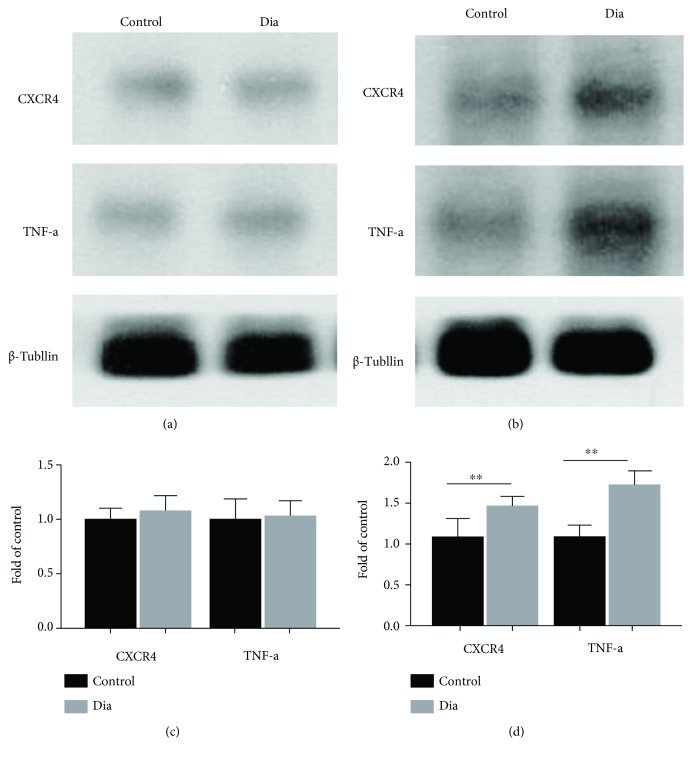
STZ-induced DNP increased the spinal cord dorsal CXCR4 and TNF-*α* of spinal cord dorsal horn at 5 weeks but not at 2 weeks of diabetes. (a, b) CXCR4 and TNF-*α* expression in spinal cord dorsal horn, as shown by western blotting, at 2 and 5 weeks after intravenous STZ and control. (c, d) Quantification of CXCR4 and TNF-*α* in spinal cord. The western blot results are presented as means ± SD (^∗^*P* < 0.05, compared with control group, Student's *t*-test, *n* = 6/group).

**Figure 4:**
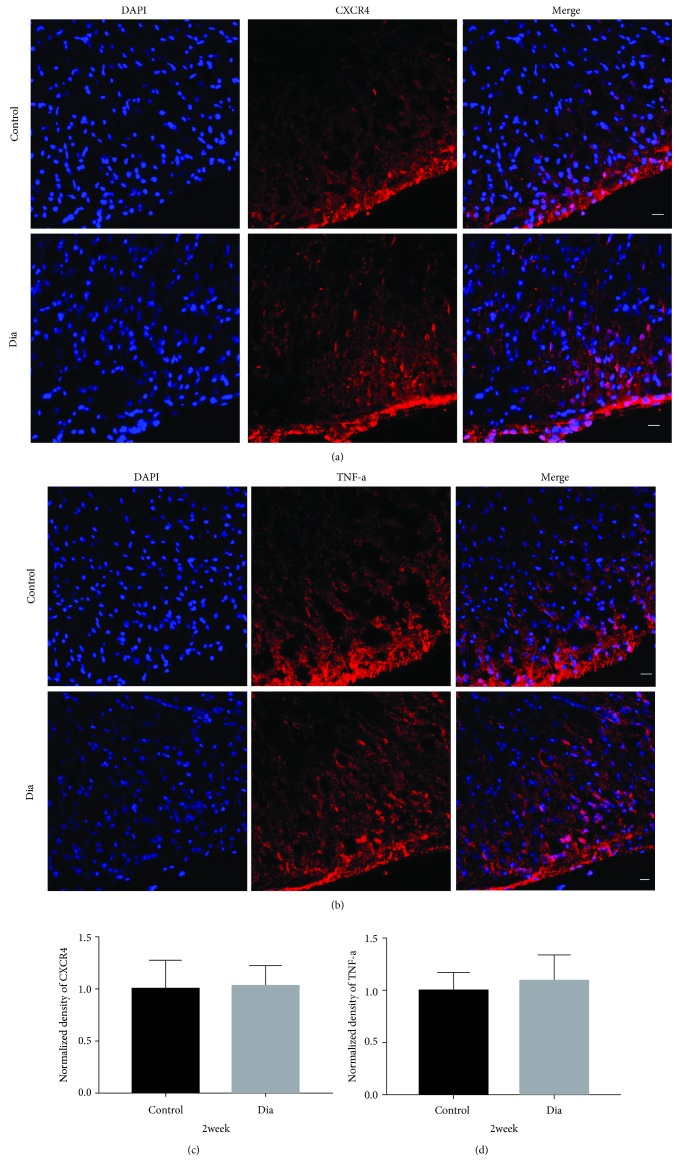
DNP did not significantly increase the expression of CXCR4 and TNF-*α* in the spinal cord dorsal horn at 2 weeks of diabetes. Confocal images in the spinal cord dorsal horn CXCR4 ((a), A-F) and TNF-*α* ((b), A-F) after intravenous STZ injection show no significant differences at 2 weeks. C and F are merged images A and B, D and E. Quantification of CXCR4 (c) and TNF-*α* 2 weeks (d) immunofluorescence intensity (*n* = 6/group. All data are presented as means ± SD).

**Figure 5:**
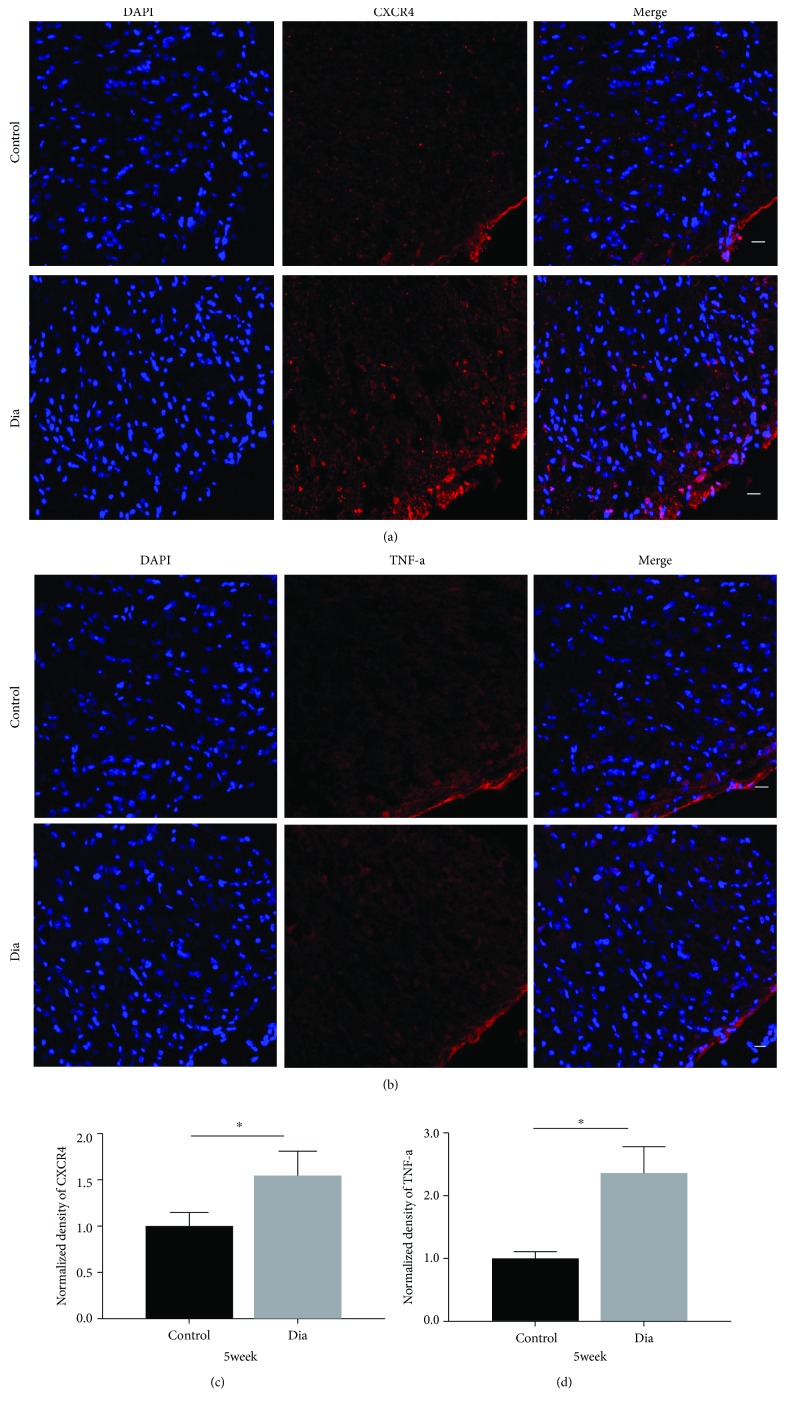
DNP significantly increased the expression of CXCR4 and TNF-*α* in the spinal cord dorsal horn at 5 weeks of diabetes. Immunostaining for CXCR4 ((a), A–F) and TNF-*α* ((b), A–F) of the spinal cord dorsal horn was measured at 5 weeks after STZ-induced diabetes. Quantitative analysis of CXCR4 and TNF-*α* of the intensity at 5 weeks. ^∗^*P* < 0.05, compared with control group. *n* = 6/group. All data are presented as means ± SD.

The spinal cord proinflammatory TNF-*α* and CXCR4 protein expressions were determined by Western blot in the control and diabetic rats with DNP. Both the spinal cord TNF-*α* and CXCR4 protein expressions were significantly increased at 5 weeks (Figures 3(c) and 3(d), *P* < 0.05) but not at 2 weeks of diabetes (Figures 3(a) and 3(c), *P* > 0.05, diabetes vs. control) despite significant reductions in the thresholds of PWT and PWL as early as 1 week of diabetes (Figure 2) as compared to control. Furthermore, spinal cord immunostaining confirmed that the immunostaining density of TNF-*α* and CXCR4 in the diabetic group did not significantly differ from that in the control group at 2 weeks of diabetes (Figures 4(a) and 4(b)), while the spinal cord immunostaining density of TNF-*α* and CXCR4 at 5 weeks of diabetes was significantly higher than that in the control group (Figures 5(a) and 5(b), *P* < 0.05).

### 3.3. Changes of DRG TNF-*α* and CXCR4 Protein Expression over Time in Diabetic Rats

**Figure 6:**
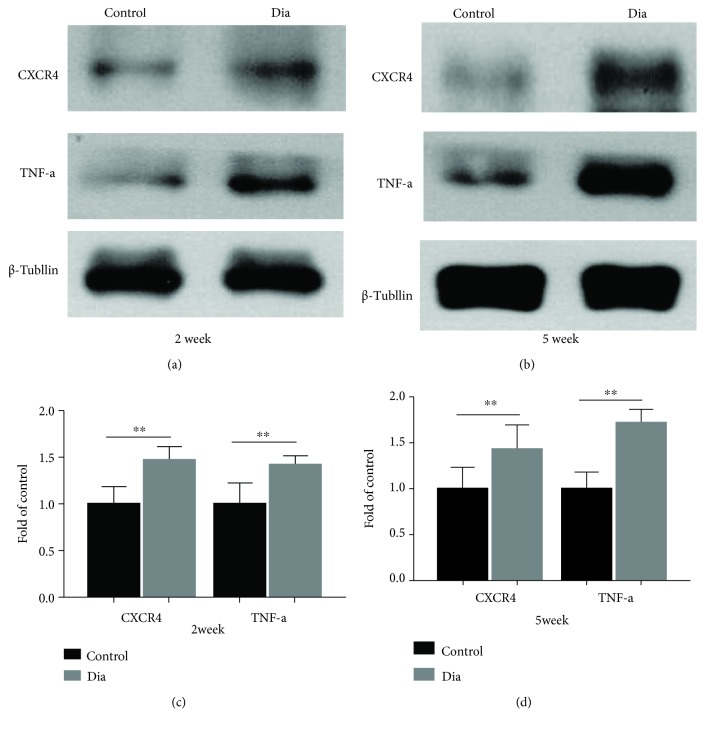
Persistent upregulation of correlative proinflammatory protein TNF-*α* and CXCR4 in the DRG in rats with DNP both at 2 weeks and at 5 weeks of diabetes. (a, b) Western blot showed the protein expression of CXCR4 and TNF-*α* in DRG at 2 and 5 weeks. (c, d) Quantitative analysis of CXCR4 and TNF-*α* in DRG at 2 weeks (c) and 5 weeks (d) of diabetes. All the data are presented as means ± SD (^∗^*P* < 0.05, compared with the control group, *n* = 6/group). DRG = dorsal root ganglion.

To look into the mechanism that might be attributable to the reduced thresholds of mechanical allodynia and heat hyperalgesia during the earlier phase of diabetes, we further investigated the status of TNF-*α* and CXCR4 protein expression over time in the DRG in addition to that in the spinal cord. As shown in Figure 6, the protein expressions of both CXCR4 and TNF-*α* were significantly increased early at 2 weeks of diabetes as compared to the control, and the DRG TNF-*α* and CXCR4 protein expression maintained significantly increased by 5 weeks of diabetes (Figures 6(a) and 6(b), *P* < 0.05, diabetes vs. control). Thus, these results illustrated that DNP induced upregulation of inflammation protein in the DRG earlier than that in the spinal cord, which may be the mechanism that is responsible for the increased sensitization to PWT and PWL during the early phase of diabetes. It suggests that persistent peripheral sensitization may induce central sensitization of the spinal cord dorsal.

## 4. Discussion

In the present study, we found that the pain threshold decreases significantly one week after the establishment of diabetes and that the numbers of rats that developed DNP in STZ-induced diabetic rats reduce over time as assessed by Von Frey and Hargreaves Apparatus. Furthermore, proinflammatory proteins TNF-*α* and CXCR4 are significantly upregulated in the spinal cord at a relatively late phase but not in the early phase of DNP. By contrast, DNP induces upregulation of TNF-*α* and CXCR4 in the DRG at both the early phase and the late phase of diabetes. These findings indicate that persistent peripheral sensitization and constant stimulation of inflammation proteins would induce central sensitization of spinal cord dorsal.

Intraperitoneal or intravenous administration of STZ is a common and simple method to produce a DNP model with one-time injection [22, 28]. It has been reported that the intravenous STZ-induced DNP model is more stable and reproducible as compared to that induced by intraperitoneal STZ injection [28]. Various injection doses of STZ have been used for the induction of diverse diabetes models [29]. STZ impairs *β*-cell in the pancreas [22, 30], and intravenous STZ at the dose of 65 mg/kg reliably and reproducibly induced type 1 diabetes in rats as we and others [18, 22, 27] established which share the properties of type 1 diabetes clinically. In the current study, the pain paw threshold was measured by electronic Von Frey and Hargreaves Apparatus before/after intravenous STZ. The measurement of mechanical allodynia was initially established early in the year of 1980 by Von Frey [31] and the mechanical allodynia has been measured by manual Von Frey ever since and being widely accepted as the golden standard of mechanical allodynia assessment. Assessment of mechanical allodynia can be performed by different methodological approaches, including the manual Von Frey, the Randall-Selitto test, and the more recently developed electronic Von Frey [32]. Electronic Von Frey has great advantages though manual Von Frey remains in measuring mechanical allodynia in that electronic Von Frey uses only a single filament to obtain continuous values over time which greatly shortens the time of measurement [33–35]. Studies showed that the values measured by electronic and manual Von Frey did not differ [35]. In the recent years, electronic Von Frey has been widely used to assess peripheral and central sensitization.

For chronic pain patients, discriminative validity of peripheral and central sensitization can be assessed by clinical manifestation [7], but the mechanism is largely unknown. Inflammation proteins and DNP are tightly correlated. STZ-induced expression upregulation of proinflammatory proteins (e.g., CXCR4 and TNF-a) of spinal cord is increased in rats [36, 37]. In the animal model of chronic constriction injury (CCI), CXCR4 expression increased in the DRG as early at day 1 to 3 and at late phase (14 day) [38]. In our study, the expression of CXCR4 and TNF-*α* in the spinal cord did not alter in rats with DNP at an early phase (<2 weeks) but increase in the DRG. While at the late phase of diabetes (i.e., 5 weeks), the protein expression of both CXCR4 and TNF-*α* all significantly increased in the spinal cord and in the DRG, which corresponds to a significant reduction of the paw withdrawal threshold and latency at 5 weeks of diabetes. Therefore, peripheral and central sensitization do not occur at the same time, but persistent peripheral inflammation would stimulate central spinal cord sensitization.

Previous studies demonstrated that the microglia in the spinal cord was activated in various models of pain (e.g., cancer pain model and nerve ligation) [39, 40], and the upregulation of spinal mRNA levels of inflammation factors (such as TNF-*α*) was detected temporally after peripheral nerve injury [40]. Moreover, injury of dorsal nerve roots has been shown to induce hyperalgesia [41]. Activation of astrocytes in the spinal cord dorsal has been shown to be attributable to the maintenance of neuropathic pain in rodents [42]. In our research, we found that the behavior of mechanical allodynia and heat hyperalgesia was not in correspondence to the expression levels of CXCR4 and TNF-*α* in the spinal cord dorsal at 2 weeks of diabetes but paralleled to the expression levels of CXCR4 and TNF-*α* in the DRG at 2 weeks of diabetes, while at 5 weeks of diabetes the enhanced expression levels of CXCR4 and TNF-*α* at both the spinal corddorsal and DRG well reflected the reduced threshold of mechanical allodynia and heat hyperalgesia. It is possible that, in STZ-induced diabetic rats with DNP, peripheral nerve injuries initially occurred that afferent axons from sensory neurons carry information of ectopic discharges from peripheral sensory receptors to the spinal dorsal horn neurons to stimulate central sensitization to pain.

It should be noted that in our current study, the pain threshold did not continue to decline with the progression of the DNP as we assumed or even remain at a fixed value, but a tendency of gradual increase toward the baseline value was manifested. Presumably, this should not be a reflection of recovery of nerve injury with the progress of diabetes, but rather it may indicate that diabetic rats developed gradually from neuropathic pain toward the reduction or even the loss of sensation to pain. Therefore, it is hard to distinguish between the reduction/loss of sensation to pain and the normal of sensation solely based on behavioral test. However, simultaneous and/or continuous assessment of changes of proinflammatory proteins CXCR4 and TNF-*α* in the spinal cord and DRG may serve to dragonize the severity of DNP. Further study is merited to explore the potential correlation of CXCR4 and central sensitization in animal models of DNP and in clinical settings.

In conclusion, data obtained from the current study provide clues to support the notion that persistent activation of inflammation proteins in the peripheral nerve would induce central sensitization. To the best of our knowledge, our current study is the first to report that in a rodent DNP model regarding the time course of peripheral sensitization and its impact on central sensitization. This study provides clues that treatments may need to target different molecules at different sites during varying durations of DNP in order to prevent the progression of pain sensitization. Further study is needed to explore the mechanisms of cell signaling pathways for CXCR4 and TNF-*α* in central sensitization and peripheral sensitization and, in particular, to observe the variation of central sensitization after the administration of peripheral sensitization to confirm whether or not persistent activation of inflammation proteins in the peripheral nerve induces central sensitization.

## Figures and Tables

**Table 1:** The analysis of mechanical allodynia and heat hyperalgesia in the diabetes group.

Statistics change	Von Frey (%)			Thermal (s)		
Week	<40% (*n*)	40%-78% (*n*)	Total (*n*)	<5.9 s (*n*)	5.9-9.8 s (*n*)	Total (*n*)
1	5 (26.3%)	12 (63.1%)	17 (89.5%)	2 (10.5%)	14 (73.7%)	16 (84.2%)
2	2 (11%)	9 (47.4%)	11 (57.9%)	1 (5.3%)	9 (47.4%)	10 (52.6%)
3	0 (0%)	9 (47.4%)	9 (47.4%)	1 (5.3%)	13 (68.4%)	14 (73.6%)
4	0 (0%)	10 (52.6%)	10 (52.6%)	4 (21.1%)	6 (31.6%)	10 (52.6%)
5	0 (0%)	8 (42.1%)	8 (42.1%)	1 (5.3%)	9 (47.4%)	10 (52.6%)

The number statistics of diabetic neuropathic pain was analyzed after intravenous STZ rats in various time courses. 40% and 78% correspond to the average pain threshold at the+ first week minus or plus the SD of Von Frey test, respectively. Similarly, 5.9 s (second) and 8 s correspond to the average pain threshold at the first week minus or plus the SD of the Hargreaves test. SD = standard deviation.

## Data Availability

The functional and biochemical data used to support the findings and the conclusion of this study are available from the corresponding author upon request.
